# Guided bone regeneration using 1-ethyl-3-(3-dimethylaminopropyl) carbodiimide (EDC)-cross-linked type-I collagen membrane with biphasic calcium phosphate at rabbit calvarial defects

**DOI:** 10.1186/s40824-015-0038-y

**Published:** 2015-07-30

**Authors:** Jin-Young Park, Im-Hee Jung, You-Kyoung Kim, Hyun-Chang Lim, Jung-Seok Lee, Ui-Won Jung, Seong-Ho Choi

**Affiliations:** Department of periodontology, Research institute of periodontal regeneration, Yonsei University College of Dentistry, 50 Yonsei-ro Seodaemun-gu, Seoul, 120-752 Republic of Korea; Department of Dental hygiene, College of Health Sciences, Eulji University, Seong-nam, Republic of Korea

**Keywords:** Guided bone regeneration, Cross-linked collagen membrane, 1-ethyl-3-(3-dimethylaminopropyl) carbodiimide

## Abstract

**Background:**

*In-vitro* and animal studies using EDC cross-linked membranes have shown great resistance to enzymatic digestion as well as low cytotoxicity, and indicated its potential expediency as a barrier membrane for guided bone regeneration (GBR). The purpose of this study was to evaluate the efficacy, biocompatibility and degradation kinetics of a novel 1-ethyl-3-(3-dimethylaminopropyl) carbodiimide (EDC)-cross-linked type I collagen membrane for regeneration of rabbit calvarial defects. EDC cross-linked type I collagen membrane and macroporous biphasic calcium phosphate (MBCP) consisting of 60 % hydroxyapatite and 40 % β-tricalcium phosphate were used in this study. Four circular defects (ø = 8 mm) were created in each calvarium of 12 male white rabbits. The experimental groups randomly allocated to the defects were as follows – (1) sham control, (2) EDC-cross-linked collagen membrane (EDC membrane), (3) bone graft (BG), and (4) bone graft with collagen membrane (B-EDC membrane). Specimens were harvested at 2 weeks (*n* = 6) and 8 weeks (*n* = 6) postoperatively for observational histology and histometrical analysis.

**Result:**

The histologic observation showed close adaptation of the EDC membrane to the defect perimeters along with vascularization of the membrane at 2 weeks. Direct apposition of new bone on to the collagen matrix could be observed displaying adequate tissue integration. Collapsing of the central portion of the membrane could be seen in the EDC membrane group, and both BG and B-EDC membrane groups showed greater total augmented area and new bone area than the EDC membrane group. The membrane was largely unresorbed at 2 weeks; and at 8 weeks the overall shape of the membrane was still maintained suggesting sustained barrier function at 8 weeks.

**Conclusion:**

Within the limits of this study, it may be concluded that EDC-cross-linked collagen membrane is a safe biomaterial with adequate tissue integration and resorption kinetics to support bone regeneration when used in conjunction with bone filler.

## Background

Guided Bone Regeneration (GBR) involves the surgical placement of barrier membranes in order to create and maintain a secluded space to promote osseous proliferation [1–4]. The ideal barrier membrane must be biocompatible, cell-occlusive, integrated by the host tissues, clinically manageable and able to uphold space [1, 5]. Although expanded polytetrafluoroethylene (e-PTFE) had been accepted as the gold standard material, susceptibility to exposure with subsequent progression of infection as well as the need for second surgical intervention had led to development of resorbable membranes. A resorbable membrane that would allow transmembraneous angiogenesis, but effectively exclude undesired cells from the clot would have the advantage of not requiring surgical removal.

Collagen membranes have advantages related to biological properties of collagen itself such as minimal inflammation and cytotoxicity, ability to facilitate cellular growth [6, 7], hemostatic ability, allowing early wound stabilization, semipermeability allowing nutrient passage, and chemotactic ability to attract fibroblasts [8]. Furthermore, collagen membranes are mechanically malleable, adaptable, and easy to manipulate during clinical procedures. Successful GBR using native collagen membranes is well-documented in the literature [2–4, 9–13]. However, the disadvantage of native collagen is inherent in the duration of barrier function which cannot be strictly controlled where loss of structural integrity and solubilization via phagocytosis may precede the completion of healing [14]. Similarly, early removal of e-PTFE membranes have been reported to resulted in reduced bone formation and incomplete bone fill [15, 16]. Hence in order to reinforce collagen membrane, various methods have been introduced to cross-link collagen. [12, 17, 18]. Several *in vivo* studies have demonstrated that cross-linked collagen membranes display prolonged membrane integrity compared with the non-cross-linked membranes [11, 12].

Although cross-linking of collagen is a commonly used procedure, its impact on physicochemical properties of the membrane is unknown. Previous studies have reported increase in perforation incidence at cross-linked collagen membrane sites compared to non-crosslinked membrane sites [12, 14]. Increasing degrees of cross-linking have indicated compromise in biocompatibility of the membrane. Rothamel et al. have reported the tendency of cross-linked collagen membrane to split from adjacent connective tissues as well as lack of vascularization in the early healing phase and foreign body reactions in three differently cross-linked collagen membranes in rat [19]. Crosslinking agents such as ultraviolet radiation, glutaraldehyde, diphenyl-phosphorylation-azide have been reported to induced inflammatory reactions and failure to integrate with host tissues. Nevertheless, certain cross-linked collagen membrane using polysaccharide has shown a degree of success for bone regeneration. Furthermore, Zubery et al. have reported osseous integration of ribose cross-linked collagen membrane in the canine jaw and humans [4, 20].

1-ethyl-3-(3-dimethylaminopropyl) carbodiimide (EDC) has widely been used in cross-linking of collagen in biomedical materials. However, to the best of our knowledge, publications in EDC-cross-linked collagen membrane for GBR procedure are scarce. *In-vitro* and animal studies using EDC-cross-linked porous membranes have shown great resistance to enzymatic digestion as well as low cytotoxicity, and have indicated its potential expediency as a scaffold in tissue engineering [6, 21, 22]. Recently, a new EDC-cross-linked collagen membrane has been developed for use in GBR and guided tissue regeneration (GTR). This membrane apparently has resilience to enzymatic degradation, biocompatibility, cell-occlusiveness, space maintenance and ideal modulus of elasticity for surgical manipulation. In a preceding study, Lee et al. investigated this collagen membrane [23], and found minimal surface resorption had occurred after 4 weeks of healing, and new bone had ossified beneath the membrane suggesting high bio-compatibility between the membrane and osteblasts [3, 23].

The aim of this study was to investigate the EDC-cross-linked type-**I** collagen membrane for the regeneration of rabbit calvarial defects at 2 and 8 weeks. Histological samples were examined to evaluate the biocompatibility, efficacy and degradation kinetics of the EDC-cross-linked type-I collagen membrane. Synthetic bone graft particles consisting of 60 % hydroxyapatite and 40 % β-tricalcium phosphate were used in combination with the barrier membrane in this study, as addition of bone grafts or space provision agents has been regarded essential for successful GBR [24, 25].

## Materials and methods

### Collagen barrier membrane

**Fig. 1:**
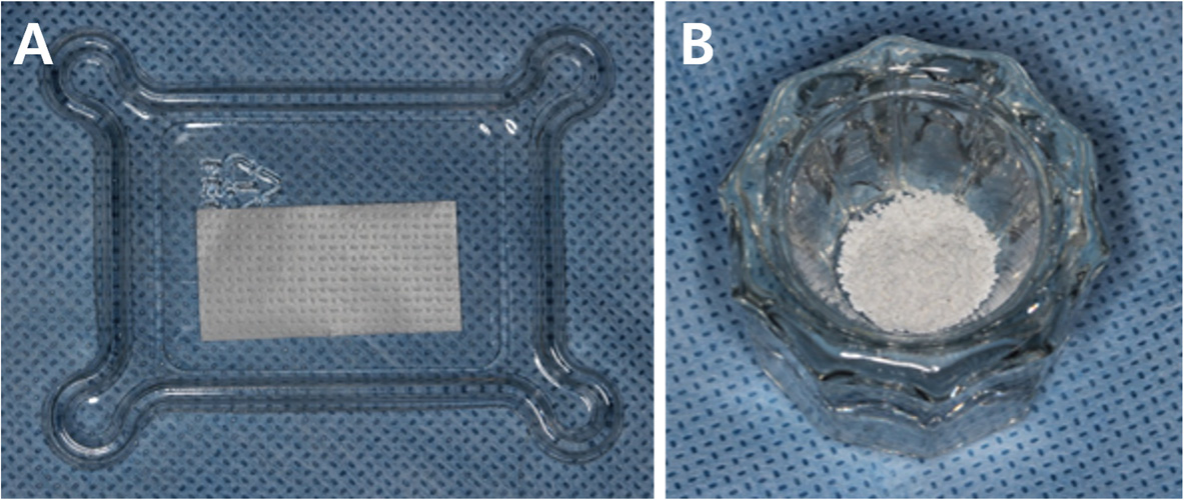
Collagen membrane and biphasic calcium phosphate. (**a**) A novel EDC crosslinked collagen membrane (Dalim Tissen©) was applied over the defects. (**b**) Biphasic calcium phosphate particles (MBCP) consisting of 60 % hydroxyapatite and 40 % β-tricalcium phosphate were used

A commercially available collagen membrane cross-linked using 1-ethyl-3-(3-dimethylaminopropyl) carbodiimide (EDC) was used (Rapigide, Dalim Tissen, Korea) (Fig. 1a). The collagen membranes were immersed in a 5 mM EDC solution for 24 h at 4 °C for cross-linking, followed by thorough washing and re-lyophilization.

### Bone graft

Macro–micro porous biphasic calcium phosphate granules (MBCP™) consisting of 60 % hydroxyapatite and 40 % β-tricalcium phosphate was used (Biomatlante, France) (Fig. 1b). The granules are 1-2 mm in size with 70 % porosity.

### Animals

Twelve male New Zealand White rabbits weighing 2.8-3.2 kg and aged 16–20 weeks were used in this study. Animal selection and management, surgical protocol, and preparation followed routines approved by the Institutional Animal Care and Use Committee, Yonsei Medical Center, Seoul, Korea.

### Study design

**Fig. 2:**
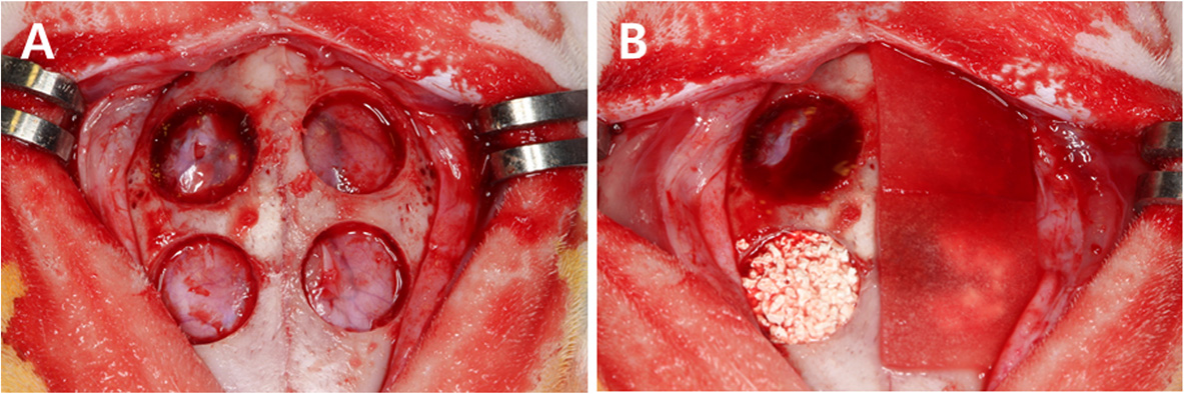
The surgical procedure. (**a**) Four circular defects (ø = 8 mm) were created in each calvarium of 12 male white rabbits, and four groups were randomly assigned to the defects. (**b**) Random assignment of the defects clockwise from top left - control group, collagen membrane only (CM), bone graft with collagen membrane (B-CM) and bone graft only (BG). Specimens were harvested at 2 and 8 weeks postoperatively for histologic and histometric analysis

Four defects of 8 mm in diameter were created in each animal. The depth of the defects comprised full thickness of the calvarial bone, with slight variation in thickness according to individual specimen and location within the calvarium. Therefore the defects were randomly assigned to the following 4 groups: empty control, EDC-cross-linked collagen membrane (EDC membrane group), bone graft (BG group), and bone graft with EDC-cross-linked collagen membrane (B-EDC membrane group). The animals were euthanized at either 2 or 8 weeks postoperative (Fig. 2).

### Surgical protocol

General anaesthesia was induced in all animals using intramuscular injections of zoletil (15 mg/kg) and rompun (5 mg/kg). The head of the rabbit was shaved and disinfected using Povidone iodine prior to local anaesthetic injections at the surgical site using 2.2 ml lidocaine hydrochloride 2 % with adrenaline 1:80,000. An incision was made along the midline of the cranium from the frontal bone to the occipital bone in order to expose the entire calvarium. A full-thickness flap was elevated. Under copious saline irrigation, four standardized round defects, each 8 mm in diameter, were created using a trephine bur. The resected bone windows were removed carefully to avoid injury to the underlying brain tissue. The four experimental groups described above were randomly applied to the defects created. For the groups containing bone graft, the amount of graft particles were standardized to completely fill each defect by application of gentle pressure using a surgical instrument. For the groups containing the barrier membrane (i.e. EDC membrane and B-EDC membrane groups), the membrane was cut to the size of 10 × 10 mm to cover the entire perimeter of each defect. The flaps were repositioned and sutured with a resorbable suture material. The animals were sacrificed at either 2 weeks (*n* = 6) or 8 weeks (*n* = 6) postoperative. The skin flaps were then reflected and the entire calvarium was harvested from each animal.

### Histologic processing

Block sections of the surgical sites were fixed in 10 % formalin for 10 days. The fixed specimens were decalcified in 5 % formic acid for 14 days and then embedded in paraffin. Serial 5 μm thick sections were cut through the central portion of each experimental site. Only sections located at the middle of the defects were selected, and stained with hematoxylin-eosin and Masson Trichrome for histologic observation and histomorphometric analysis.

### Clinical observations

Animals were carefully observed for inflammation, allergic reactions, and other complications around the surgical site throughout the 2 and 8 week healing periods. The specimens were also inspected at the time of sacrifice once the calvarial bone including the experimental sites were harvested from the animal.

### Histological observations

The specimens were examined under a microscope (DM LB, Leica Microsystems, Wetzlar, Germany) equipped with a camera (DC300F, Leica Microsystems, Wetzlar, Germany) by one blinded examiner. Images of the slides were acquired and saved as digital files.

### Histomorphometric analysis

After the conventional microscopic examination, computer-assisted histometric measurements in the calvarial defect were performed using an automated image analysis system (Image-Pro Plus; Media Cybernetics, Silver Spring, MD). The following parameters were measured from each histologic section of the defect areas.

- Remaining membrane area (RM) – Area of the barrier membrane remaining within the defect.
- New bone area (NB) – Area of newly formed bone within the defect.
- Total augmented area (TA) – Total area contained within the CM or periosteum superiorly, lateral boundaries of the defect and the dura matter inferiorly. This consists of the sum of the area of new bone, residual particles, connective tissue, adipose tissue and blood vessels within the defect.
- Residual particle (RP) – Area of the remaining bone graft particles within the defect.

### Statistical analysis

The statistical analysis was performed using a commercially available software program (SPSS 18.0, SPSS, Chicago, IL). Histomorphometric records from the calvarial defect samples were used to calculate the mean and standard deviation (SD) values of the four groups (i.e. control, BG, CM, B-CM). Kruskal Wallis test and Mann-Whitney *U* test (nonparametric analysis of variance) was used to analyze the difference between the groups at each time periods, and also to compare the same experimental group between the two healing periods. Statistical significance was considered when *P* < 0.05.

## Results

### Clinical observations

Healing was uneventful, and no signs of adverse inflammatory reaction or complications were observed at the surgical sites prior to sacrifice. At visual inspection of the surgical sites during harvesting, primary closure of the flaps was confirmed and no exposure of the membranes through the overlying periosteum was noticed. All experimental sites remained intact. The defect spaces appeared encapsulated by periosteum and dura mater, preventing discharge of any defect constituents such as the bone graft particles.

### Histologic observations

#### Control group

**Fig. 3:**
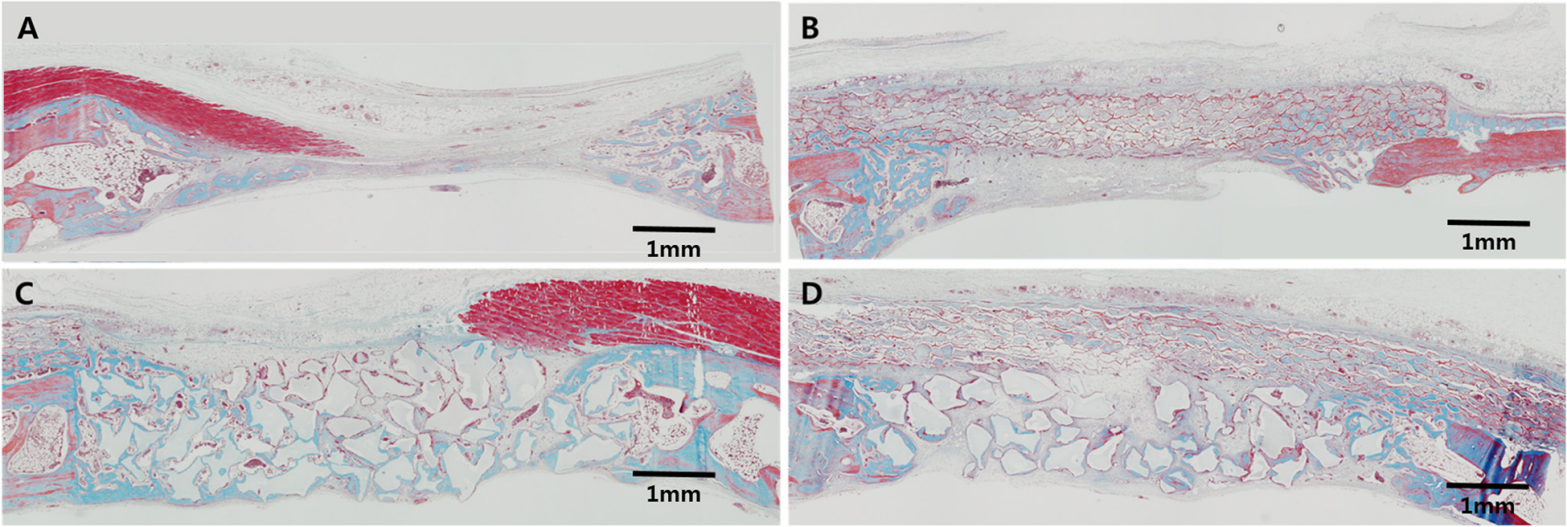
Histologic view after 2 weeks of healing, showing – (**a**) control group; defect is mainly occupied by the overlying soft tissues that had collapsed into the space. (**b**) Collagen membrane group; membrane appeared structurally intact and displayed close marginal adaptation to the native bone at the perimeter of the defects. (**c**) Bone graft group; defect space is well-maintained compared to the EDC group. (**d**) Bone graft with collagen membrane group; defect space is well-maintained by the barrier membrane and the supporting bone graft particles. *X40 magnification view, Trichrome staining*

At 2 weeks, woven bone structures were observed occurring from the defect margins (Fig. 3a). The defects were mainly occupied by the overlying soft tissues that had collapsed into the space. The periosteum appeared to contain many blood vessels. Infiltration of inflammatory cells, mainly macrophages, as part of the normal healing sequence could be observed in even distribution throughout the experimental site.

**Fig. 4:**
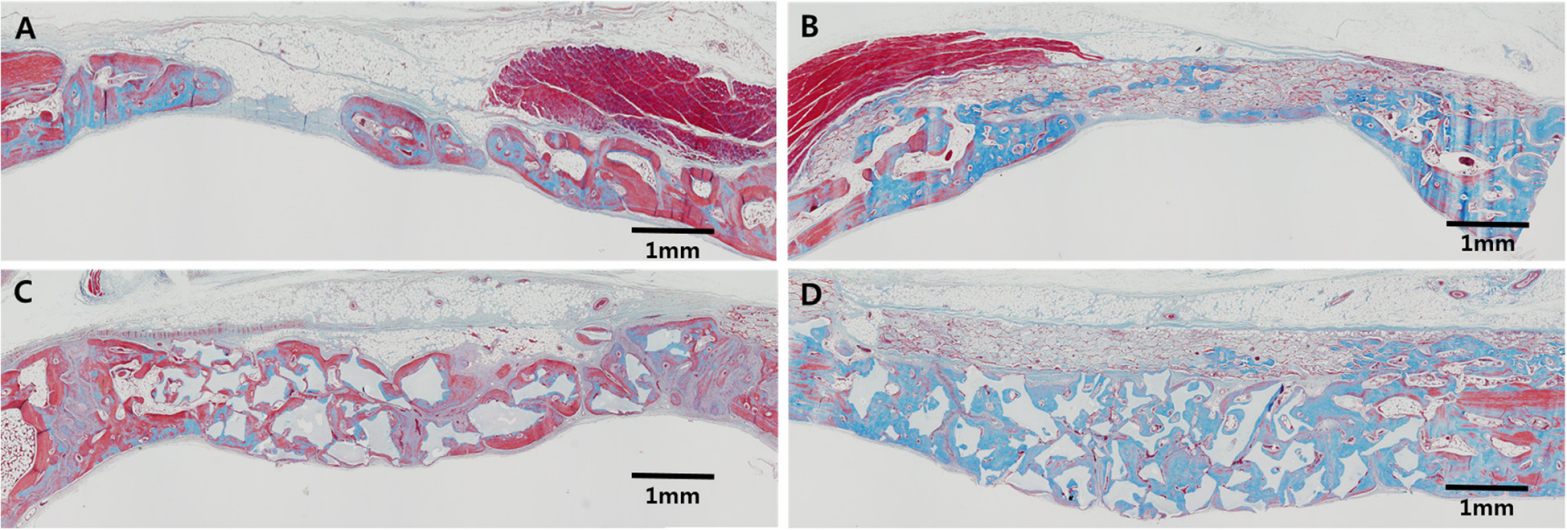
Histologic view after 8 weeks of healing showing – (**a**) control group; defect spaces were filled by the overlying soft tissues and dense fibrous tissues. (**b**) Collagen membrane group; the collagen membrane showed clear reduction in structural integrity as resorption had taken place. New bone was integrated with the collagenous network of the membrane and gradually occupied the space in which the membrane existed previously. (**c**) Bone graft group; Mature new bone formation could be observed. The superficial layer of bone graft particles were surrounded by fibrous tissue and the regenerated bone was slightly below the original height of native bone. (**d**) Bone graft with collagen membrane group; new bone surrounded the bone graft particles, which were interconnected to form complete closure of the defect space. New bone formation extended from the native bone at the defect perimeter into the covering membrane that was closely adapted to the native bone. *X40 magnification view, Trichrome staining*

At 8 weeks, formation of bony islands were seen on central areas of the defect (Fig. 4a), which can be construed to be the products of osteogenic activity from the surrounding periosteum and dura mater [26]. Almost full closure along the diameter of the defect could be seen on certain histological sections; however the height of the restored bone was far below the level of the native bone. The remaining defect spaces were filled by the overlying soft tissues and dense fibrous tissues. A clear decrease in the number of inflammatory cells could be observed compared to the histologic view at 2 weeks (Fig. 4a).

#### EDC membrane group

At 2 weeks, the collagen membrane appeared structurally intact and displayed close marginal adaptation to the native bone at the perimeter of the defects. Formation of blood vessels could be seen around the collagen membrane in close proximity to the membrane (Fig. 3b). Collapsing of the collagen membrane at the central region in addition to the elevation of the underlying dura mater resulted in the subsequent elimination of majority of the defect space. Wedge-shaped woven bone structures protruded from the perimeter of the defects. The new bone growths towards the central direction were confined by the intruding barrier membrane. Appearance of inflammatory cells, mainly macrophages, as part of the wound healing sequence could be seen at the experimental site. The macrophages were seen to be gathered in concentrated clusters distributed in and around the implanted materials.

**Fig. 5:**
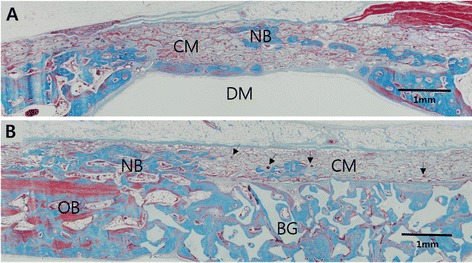
Higher magnification view of the defect sites at 8 weeks, Trichrome staining. (**a**) Histologic view of CM group showing islets of new bone (NB) integrated within the CM at the middle of the defect. Depression of the collapsed CM and elevation of the dura mater (DM) resulting in subsequent elimination of defect space. Nevertheless, new bone (NB) formation occurred integrated within the CM. (**b**) Histologic view of collagen membrane (CM) in the B-CM group showing new bone (NB) integrated to the network of collagen membrane. CM is closely adapted to the native bone (OB) at the defect perimeter. Effective space provision is provided by the bone graft (BG) particles. Vascularization of the membrane can be seen throughout the membrane (arrows)

**Fig. 6:**
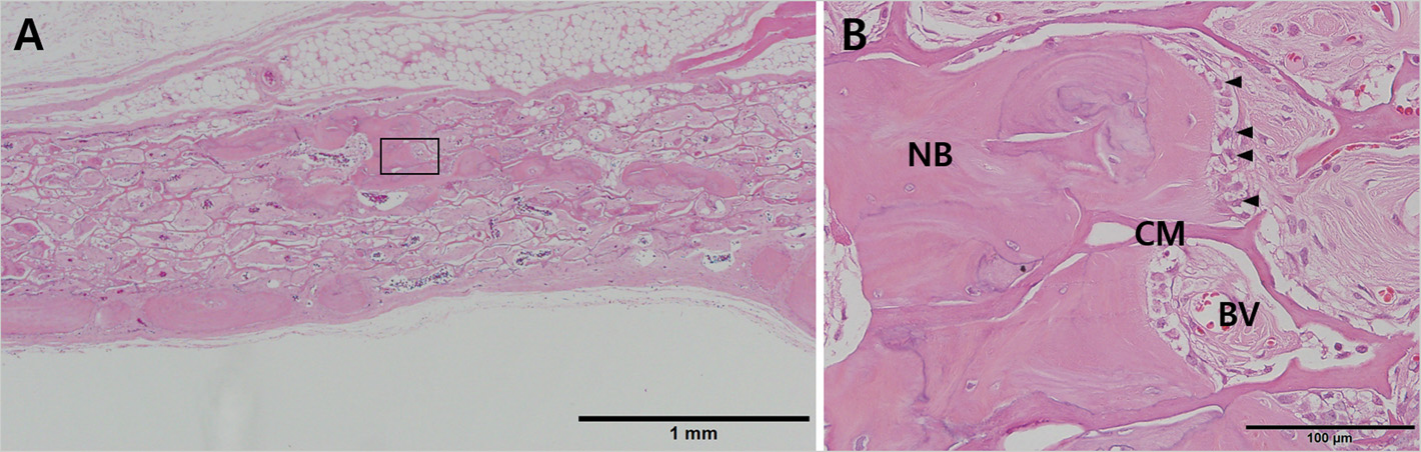
Bone formation around EDC membrane at 8 weeks. (**a**) EDC membrane group at 8 weeks showing mature new bone formation within the membrane space. *X40 magnification, Hematoxylin & eosin staining.*(**b**) Formation of new bone matrix (NB) by the osteoblasts (arrows) can be seen. New bone is appeared to be maturing into lamellar structures as seen by the appearance of osteocytes and the resulting Haversian system. Newly formed bone is in contact with the membrane structures (CM), which suggests good biocompatibility and tissue integration of the EDC membrane. Formation of transmembraneous blood vessels (BV) can also be seen occurring within the membrane space. *X400 magnification, Hematoxylin & eosin staining*

At 8 weeks, the collagen membrane appeared to maintain the original thickness and shape seen at 2 weeks (Fig. 4b). Mature new bone with osteocytes was formed mainly at the perimeters and at the central areas as bony islands (Figure 5a). New bone was integrated with the collagenous network of the membrane and gradually occupied the space in which the membrane existed previously (Figs. 4b and 6). At high magnification, secretion of new bone matrix by osteoblasts could be observed within the collagen membrane, and the secreted new bone was fully integrated with the membrane. Furthermore, formation of blood vessels could be seen within the membrane space (Fig. 6b). More sparse distribution of inflammatory cells could be observed with clear reduction in numbers compared to 2 weeks.

#### BG group

At 2 weeks, defect space was well-maintained compared to the EDC membrane group (Fig. 3c and b). The bone graft particles formed an irregular margin against the overlying soft tissues with encapsulation by the fibrous tissues superiorly (Fig. 3c). Early new bone formation could be observed, clustered around each bone graft particle as well as from the native bone at the perimeters. In comparison to the B-EDC membrane group, fewer graft particles appeared to be present in the BG group. Inflammatory cells appeared in similar appearance to the EDC membrane group distributed in concentrated clusters around the implanted materials.

At 8 weeks, lamellar bone structures with osteocytes could be observed; however, fibrous tissues still occupied areas inside the defect (Fig. 4c). The superficial layer of bone graft particles were surrounded by fibrous tissue and the new bone was restored slightly below the original height of native bone. Little resorption of the bone graft particles appeared to have taken place at 8 weeks compared to 2 weeks. (Figs. 4c and 3c).

#### B-EDC group

At 2 weeks, early new bone appeared mainly around particles at the base of the defect. Blood vessels formation was noticed within the collagen membrane (Fig. 3d). The defect space was well-maintained by the barrier membrane and the supporting bone graft particles. The augmented areas were often greater than the original bone area as packing of the bone graft particles during the surgical procedure expanded the defect space by exerting pressure onto the underlying dura mater. Inflammatory cells were again found in similar appearance to the other experimental groups, forming small concentrated clusters around implanted materials.

At 8 weeks, new bone surrounded the bone graft particles, which were interconnected to form complete closure of the defect space (Fig. 4d). New bone formation extended from the native bone at the defect perimeter into the covering membrane that was closely adapted to the native bone (Figure 5b). Clear reduction in the area of the membrane could be seen; however, little resorption of the graft particles had taken place compared to 2 weeks. More sparse distribution of inflammatory cells could be observed with clear reduction in numbers compared to 2 weeks.

### Histomorphometric analysis

The results from the histomorphometric analysis are listed on Table 1.

**Table 1:** Histomorphometric analysis at 2 and 8 weeks postoperative

	Group	Total augmented area	Residual particle	New bone	Remaining membrane
2 weeks	Control	4.21 ± 1.52	-	0.95 ± 0.81^b^	-
	EDC	3.49 ± 1.07	-	0.95 ± 0.31^b^	5.51 ± 0.73^b^
	BG	10.04 ± 2.03^a^	2.39 ± 1.00	1.93 ± 0.61^b^	-
	B-EDC	11.49 ± 2.82^ab^	3.17 ± 1.05	1.98 ± 0.70^b^	5.58 ± 0.97^b^
8 weeks	Control	5.21 ± 1.40	-	2.76 ± 0.92^b^	-
	EDC	4.23 ± 1.00	-	2.11 ± 0.64^b^	1.98 ± 1.89^b^
	BG	12.54 ± 1.64^a^	2.70 ± 0.73	4.76 ± 1.61^ab^	-
	B-EDC	14.59 ± 3.13^ab^	2.84 ± 0.66	5.10 ± 1.68^ab^	1.47 ± 1.65^b^

Statistically significant difference from the control group

^a^Statistically significant difference from the membrane group

^b^Statistically significant difference between the same experimental group between 2 and 8 weeks

#### 2 weeks

At 2 weeks, there was no significant difference between the control group and the EDC group in terms of TA and NB. BG group and B-EDC membrane group both showed greater TA compared to the control group with statistical significance (*P* = 0.006, *P* = 0.006, respectively). In regards to the NB, although BG and B-EDC membrane groups showed greater mean NB than the control, this was not statistically significant (*P* = 0.055, *P* = 0.126, respectively). Both BG and B-EDC groups showed greater TA and NB compared to the EDC membrane group with statistical significance (TA: *P* = 0.004, 0.004, NB: *P* = 0.008, 0.037, respectively).

#### 8 weeks

At 8 weeks, there was no significant difference between the control group and the EDC membrane group in terms of TA and NB. BG group and B-EDC membrane group both showed greater TA compared to the control group with statistical significance (*P* = 0.006, *P* = 0.006, respectively). In regards to mean NB, both BG and B-EDC membrane groups were greater than the control group with only the B-EDC membrane group showing statistically significant difference (*P* = 0.018). Both BG and B-EDC groups showed greater TA and NB than the EDC membrane group with statistically significant difference (TA: *P* = 0.004, 0.004, NB: *P* = 0.010, 0.004, respectively).

#### Between 2 & 8 weeks

In all 4 groups, statistically significant difference was shown in regards to NB between 2 and 8 weeks (control: *P* = 0.016, EDC membrane: *P* = 0.010, BG: *P* = 0.010, B-EDC membrane: *P* = 0.004).

The RM had decreased from 2 to 8 weeks, displaying 68 % mean resorption over 6 weeks. This difference was statistically significant in both EDC membrane groups and B-EDC membrane groups between 2 and 8 weeks (*P* = 0.010, *P* = 0.004, respectively).

The RP of both BG and B-EDC membrane groups between 2 and 8 weeks showed no difference statistically. This result concurs with the histological observation in which no visible reduction in RP could be detected.

## Discussion

1-ethyl-3-(3-dimethylaminopropyl) carbodiimide (EDC) has widely been used in cross-linking of collagen in biomedical materials. In-vitro and animal studies using EDC cross-linked membranes have shown great resistance to enzymatic digestion as well as low cytotoxicity, and indicated its potential expediency as a barrier membrane for guided bone regeneration (GBR). The EDC membrane in this study showed prolonged duration of function shown by the maintenance of the overall outward shape at 8 weeks. In addition, despite recent reports of compromised biocompatibility in cross-linked collagen membranes, the EDC membrane displayed remarkable tissue integration with minimal immune reactions as observed clinically and histologically. The observations of the current study has demonstrated that increased bio-durability of collagen membranes can be achieved with favorable adaptation to the surrounding tissues.

Tissue integration and biocompatibility are important criteria to fulfill for resorbable barrier membranes in order to minimize immunological response and membrane exposure. This is more relevant for the cross-linked variety as degradation product of the cross-linking agent have been shown to cause foreign body reactions. For instance, increasing degrees of cross-linking have been associated with reduced biocompatibility through *in vitro* cultures [27]. Cross-linking of collagen was shown to be associated with decreased tissue integration and vascularization. Certain enzymatic and chemical cross-linked collagens were linked with increased cytotoxicity [28]. Similarly, in a human study, a cross-linked collagen membrane with prolonged resorption time demonstrated significantly more adverse events and less bone regeneration compared to the native collagen membrane [14]. On the other hand, certain cross-linked collagen membranes using polysaccharides has been shown to be favorable for GBR procedures despite the findings of spontaneous early exposures [4, 17, 20]. Although exposure of collagen membranes to the oral environment has been reported to cause disintegration of the membrane [11], Sela et al. have demonstrated in this case that cross-linked CMs were more resistant to proteolysis than the native CM [29]. Unlike the previously studied cross-linked CMs, the current membrane did not produce soft tissue dehiscence in any of the surgical sites, and wound healing occurred without complication. Histologically, the membrane appeared to be well-integrated to the native bone with formation of vascular network throughout the defect sites; transmembraneous angiogenesis has been shown to play a crucial role in early new bone formation in the native CM [30, 31].

The aim of this study was to investigate the EDC-cross-linked collagen membrane (EDC-CM) for GBR procedure in the rabbit calvarial defect model. The calvarial defect has spontaneous healing capacity due to its contained morphology bounded by tissues encompassing osteogenic capacity such as the native bone, periosteum and dura mater. In the present study, 8 mm-diameter defects were used, which is less than the critical-size demonstrated by Sohn et al. [26]. Consequently, all of the groups including the control showed statistically significant increase in NB at 8 weeks compared to 2 weeks. However, the current experimental model is ideal for comparison of multiple experimental groups, and inclusion of a sham control eliminates the element of spontaneous bone formation during inter-group analysis.

According to the histomorphometric analysis, there was no difference between the control and EDC membrane groups in terms of TA and NB at both 2 and 8 weeks. However, with the addition of bone graft particles in the BG and B-EDC membrane groups, significantly greater TA and NB were produced. Notably, this result shows that in the current experimental model, the amount of new bone formation is directly related to the space maintained by the implanted materials. The histologic observations showed that the defect spaces were not well-maintained by the collagen membranes as seen by the collapsing of the membranes at the central portions. Although the membrane appeared dimensionally stable at initial placement during surgery, histologic evidence suggests that mechanical strength is lost upon contact with surrounding soft tissues, which is a typical drawback of the collagen membranes [32]. Adequate space maintenance is provided only when defect morphology is favorable and if the bony defect cannot support itself, the membrane is prone to collapse [33, 34]. Therefore, the results of the current study demonstrate that the same principle is applied when performing GBR with EDC-CM as the native CM in that provision of support by bone grafting is essential for space-making.

In this study, synthetic bone filler consisting of macroporous biphasic calcium phosphate particles were used. Bone fillers are effective for providing support for resorbable membranes while preventing the risk of autogenous bone harvest [35]. Biphasic calcium phosphate produced minimal immune response at 2 weeks. Vascularization occurred around the particles but displayed minimal amount of resorption at 8 weeks. New bone proliferated directly onto the bone substitute particles demonstrating both osteoconductivity and osteoinductivity of the biomaterial. In the present study, comparable amounts of new bone were obtained between the BG group and B-EDC membrane group. This is due to the contained morphology of the calvarial defect in the current study. Still, less residual particles were present in the BG group sites compared to the B-EDC membrane group, and the outline of the newly formed bone at the surface appeared more orderly in the B-EDC group. Also, fibrous encapsulation of the grafted particles at the superficial aspect was visible in the BG group; whereas in the B-EDC membrane group, new bone formation occurred along the membrane surface and integrated with the collagen matrix. This observation suggests that the use of barrier membrane enhanced new bone formation by preventing the infiltration of soft tissues and stabilizing the graft particles during surgical manipulation. In the clinical situation where augmentation of alveolar bone volume is often attempted in various defect morphologies, such barrier membrane would be crucial to maximize bone formation and to prevent scattering of the graft particles.

EDC has been found to modify side-groups on proteins to make them reactive with other side-groups and to mediate the ester bond formation between the hydroxyl and carboxyl groups. In contrast to conventional chemical agents such as glutaraldehyde or polypeptides, carbodiimides do not remain as a part of that linkage but simply change to water-soluble urea derivatives that have very low cytotoxicity. At the same time, EDC cross-linked membrane has been characterized to possess great resistance to enzymatic digestion and be toxicologically acceptable [6, 21, 22]. In the present study, no clinically abnormal reactions were noticed during the observation period of 8 weeks. Histologically, aggregation of macrophages was seen on 2-week specimens around grafted materials including the membrane and the bone substitute particles. The concentration of immune cells decreased at 8 weeks suggesting that the appearance of macrophages was part of the normal healing sequence. Furthermore, close adaptation of the EDC membrane to the defect perimeters were observed along with evidence of vascularization at 2 weeks. Direct apposition of new bone on to the collagen matrix could be observed, which shows satisfactory tissue integration and biocompatibility. This is a similar finding to an observation in another study that demonstrated tissue integration of a ribose-cross-linked collagen membrane, in which ossification developed beneath the collagen membrane and new bone showed adherence to the membrane [11, 14, 20].

Space maintenance duration has been reported to be another important factor strongly influencing the outcome of GBR [36]. Previous studies using e-PTFE membrane has shown that early removal of the membrane may result in reduced bone formation. For instance, a study in dogs has shown that bone regeneration was not completed at 4 months, and has demonstrated a definite gradient from the marginal portion toward the middle zone of the defect, where the formation of primary spongiosa was still going on, particularly in the roof of the defect [36]. Therefore, several clinicians have advocated that membranes used for GBR should last longer, 6–9 months, than guided tissue regeneration (GTR). Cross-linked collagen membranes have been shown to be more resistant to in vivo degradation than non-cross-linked membranes [19, 29]. In addition, a previous report has demonstrated a negative correlation between the degree of cross-linking and resorption rate [19]. Lee et al. have shown uniform absorption and maintenance of the outward shape at 4 weeks using the EDC-CM [23]. The appearance of the membrane in the current study illustrates that the overall shape of the membrane was still maintained after 8 weeks despite of the overall area resorption of approximately 68 %. This indicates that prolonged membrane function may be provided by the EDC-CM in comparison to the native collagen membrane, which has been shown to disintegrate as early as 4 weeks.

Recent reports have claimed a certain shift in paradigm in regards to necessity of collagen cross-linking through series of studies that demonstrate compromised biocompatibility in cross-linked collagen membranes [19, 27, 28, 30, 31]. Studies have indicated that treatment outcomes are not significantly influenced by membrane resorption time but mainly dependent upon the presence or absence of membrane exposure and/or space collapse [11, 32]. A commercially available native type I and III porcine collagen membrane has been reported to exhibit ideal tissue integration and vascularization resulting in a nearly complete biodegradation 4 weeks following implantation. Nonetheless, a biocompatible cross-linked collagen membrane with a prolonged barrier function would produce optimal outcome for GBR; as shown histologically in this study, better quantity and quality of bone was formed at the superficial layer of the defect when EDC-CM was used along with bone filler. The present study agrees with the statement that cross-linking of collagen membrane increases the resistance to biodegradation, meanwhile the capacity to tissue integrate has not been compromised using the EDC-CM.

## Conclusion

Within the limits of this study, it may be concluded that EDC-cross-linked collagen membrane is a safe biomaterial with adequate tissue integration and resorption kinetics to support bone regeneration when used in conjunction with bone fillers. Further study should be conducted for direct comparison between native collagen membrane and EDC-CM. Also more challenging defect morphology for alveolar bone augmentation than the current model should be employed with longer healing period to properly test the effectiveness of EDC-CM for GBR.

## Availability of supporting data

The data sets supporting the results of this article are included in the article.
